# In Vitro Selective Growth-Inhibitory Activities of Phytochemicals, Synthetic Phytochemical Analogs, and Antibiotics against Diarrheagenic/Probiotic Bacteria and Cancer/Normal Intestinal Cells

**DOI:** 10.3390/ph13090233

**Published:** 2020-09-03

**Authors:** Tomas Kudera, Ivo Doskocil, Hana Salmonova, Miloslav Petrtyl, Eva Skrivanova, Ladislav Kokoska

**Affiliations:** 1 Department of Crop Sciences and Agroforestry, Faculty of Tropical AgriSciences, Czech University of Life Sciences Prague, Kamycka 129, 16500 Praha-Suchdol, Czech Republic; kuderat@ftz.czu.cz; 2 Department of Microbiology, Nutrition and Dietetics, Faculty of Agrobiology, Food and Natural Resources, Czech University of Life Sciences Prague, Kamycka 129, 16500 Praha-Suchdol, Czech Republic; doskocil@af.czu.cz (I.D.); salmonova@af.czu.cz (H.S.); skrivanovae@af.czu.cz (E.S.); 3 Department of Zoology and Fisheries, Faculty of Agrobiology, Food and Natural Resources, Czech University of Life Sciences Prague, Kamycka 129, 16500 Praha-Suchdol, Czech Republic; petrtyl@af.czu.cz

**Keywords:** plant compounds, diarrhea, antibacterial, anticancer, selectivity

## Abstract

A desirable attribute of novel antimicrobial agents for bacterial diarrhea is decreased toxicity toward host intestinal microbiota. In addition, gut dysbiosis is associated with an increased risk of developing intestinal cancer. In this study, the selective growth-inhibitory activities of ten phytochemicals and their synthetic analogs (berberine, bismuth subsalicylate, ferron, 8-hydroxyquinoline, chloroxine, nitroxoline, salicylic acid, sanguinarine, tannic acid, and zinc pyrithione), as well as those of six commercial antibiotics (ceftriaxone, ciprofloxacin, chloramphenicol, metronidazole, tetracycline, and vancomycin) against 21 intestinal pathogenic/probiotic (e.g., *Salmonella* spp. and bifidobacteria) bacterial strains and three intestinal cancer/normal (Caco-2, HT29, and FHs 74 Int) cell lines were examined in vitro using the broth microdilution method and thiazolyl blue tetrazolium bromide assay. Chloroxine, ciprofloxacin, nitroxoline, tetracycline, and zinc pyrithione exhibited the most potent selective growth-inhibitory activity against pathogens, whereas 8-hydroxyquinoline, chloroxine, nitroxoline, sanguinarine, and zinc pyrithione exhibited the highest cytotoxic activity against cancer cells. None of the tested antibiotics were cytotoxic to normal cells, whereas 8-hydroxyquinoline and sanguinarine exhibited selective antiproliferative activity against cancer cells. These findings indicate that 8-hydroxyquinoline alkaloids and metal-pyridine derivative complexes are chemical structures derived from plants with potential bioactive properties in terms of selective antibacterial and anticancer activities against diarrheagenic bacteria and intestinal cancer cells.

## 1. Introduction

The lack of an effective and safe antimicrobial therapy for diarrheagenic bacterial infections is a global health concern, especially in developing countries for children under the age of five years [1]. Although mortality associated with bacterial diarrhea is low in developed countries, the increased incidence rates of inflammatory bowel disease and colorectal cancer have also been attributed to gut dysbiosis that can result from chronic intestinal infections [2]. Currently, infectious diarrhea is treated using conventional drugs belonging to various classes of antibiotics, such as ceftriaxone, chloramphenicol, ciprofloxacin, tetracycline, metronidazole, and vancomycin. However, the irrational use of antibiotics, including incorrect dose prescription, has led to the development of drug resistance in several pathogens. Additionally, the applications of conventional antibiotics are limited, especially among children in developing countries, owing to high cost and increased risk of side effects including gut dysbiosis. Therefore, there is a need to identify novel antimicrobial agents for infectious bacterial diarrhea to overcome the limitations of conventional antimicrobial drugs [3].

Over the last decades, plant-derived products have become a mainstay in providing novel chemical scaffolds for the development of anti-infective drugs, and therefore antidiarrheal medicinal plants and their bioactive components can be examined first [4]. However, the therapeutic effect of products derived from antidiarrheal medicinal plants is not necessarily based on their antimicrobial activity against the causative agents as other mechanisms can be considered important, such as antimotility and antisecretory effects, for their useful applications [5]. The chance of finding a new plant-derived compound with promising antibacterial activity could therefore be enhanced by the chemotaxonomic approach by examining such novel medicinal plants that are taxonomically related to the species known to bear specific types of phytochemicals with growth-inhibitory effect on diarrheagenic bacteria [6]. In a recent review paper, Kokoska et al. [7] described the in vitro antimicrobial properties and clinical efficacy of plant-derived compounds with their synthetic analogs that are present in products available in the international market as over-the-counter pharmaceuticals, dietary supplements, and herbal medicines for intestinal infections. Benzylisoquinoline alkaloid berberine (e.g., *Hydrastis canadensis*), simple phenol bismuth subsalicylate, the analog of salicylic acid derived from salicin (*Salix alba*), and polyphenol tannic acid (e.g., *Quercus* spp.) were mentioned as examples of the efficient agents with potent activity against some of the gut bacterial pathogens.

As it has recently been proposed, investigations of new antimicrobial agents should be focused on the identification of structures with lowered toxicity to indigenous intestinal microbiota including probiotic bacterial strains. Since there is a common association between dysbiosis and the use of antibiotics, the agents selectively acting against pathogenic microorganisms can prevent the risk of developing diseases, such as chronic bowel inflammation and intestinal carcinoma [2]. Although the composition of the whole gut microbiota comprises a large number of microorganisms forming a complex ecosystem, a preliminary screening of selective antibacterial activity of newly tested agents could be performed by testing the in vitro susceptibilities of particular representatives of each of the three dominant bacterial phyla that can be found in human intestines, and which probiotic function have been described. Among them, we can recognize the strains such as *Bifidobacterium* spp. (Actinobacteria), *Lactobacillus* spp. (Firmicutes) and *Bacteroides fragilis* (Bacteroidetes) [8,9]. Selective in vitro growth inhibitory effect of a plant-derived compound was for example described in the study of Novakova et al. [10], where anticlostridial effect of 8-hydroxyquinoline (*Microstachys corniculata*) was comparably higher than the activities revealed against different strains of bifidobacteria. Chloroxine, the synthetic 8-hydroxyquinoline derivative, is the antimicrobial agent that has also been used as an oral formulation for infectious diarrhea, and disorders of the intestinal microbiota [11]. In dysbiosis, the increased abundance of *Fusobacterium nucleatum* and *Faecalibacterium prausnitzii* is positively and negatively correlated with the risk of intestinal carcinogenesis, respectively [12]. The repression of gut microbiota enhances the susceptibility of host intestinal cells to diarrheagenic bacterial infection and chemical-induced cytotoxicity. Thus, novel antimicrobial agents should not exhibit cytotoxic activity against normal intestinal cells [13]. For example, quinolone antibiotics were reported to inhibit the growth of both bacterial and eukaryotic cells through the same mechanism and consequently enhance the risk of eliciting cytotoxic response [14,15]. On the other hand, the antiproliferative activity of these agents with selective cytotoxic effects against intestinal cancer cells would be a suitable feature in cases of dysbiosis associated with carcinogenesis. There are limited studies on the anticancer effects of conventional antibiotics. For example, Bourikas et al. [16] reported that ciprofloxacin is potent to inhibit the proliferation of intestinal cancer cell line HT29 in vitro. On the other hand, a strong in vitro antiproliferative activity of plant-derived compounds against various cancer cell lines has extensively been reported, and some are used as a scaffold for anticancer drugs. For example, the alkaloid camptothecin, extracted from the bark of *Camptotheca acuminata*, is currently used as a cytostatic agent for the treatment of colon cancer [17]. We, therefore, suggest that the phytochemicals with known in vitro growth-inhibitory activity against some of the diarrheagenic bacteria could potentially exhibit selective cytotoxic effects on cancer cells. Amongst them, the anticancer effect has generally been reported for quinoline alkaloids [18]. In addition to those earlier mentioned, benzylisoquinoline alkaloid sanguinarine (*Sanguinaria canadensis*) [7,19], and 8-hydroxyquinoline derivatives ferron [20] and nitroxoline [21] are examples of antimicrobial drugs with potent anticancer activity. Another type of synthetic phytochemical analog with reported antimicrobial and anticancer properties is metal-pyridine derivative complex zinc pyrithione (pyrithione found in *Polyalthia nemoralis*) [7,22].

In this study, we compared the selective antibacterial (diarrheagenic/probiotic strains) and cytotoxic (cancer/normal intestinal cells) activities of phytochemicals (alkaloids and phenolics) and their synthetic analogs with those of antibiotics in vitro. For each test compound, values of minimum inhibitory concentrations (MICs), half-maximal inhibitory concentration (IC_50_), and 80% inhibitory concentration of proliferation (IC_80_) were assessed. The means of these values ($\bar{x}$*-*MIC, $\bar{x}$*-*IC_50_, and $\bar{x}$-IC_80_), each defined for a particular type of strain/cell line, were used for calculation of selectivity index (SI) between activities against normal intestinal cells and diarrheagenic strains (SIa), probiotic and diarrheagenic strains (SIb), and normal and cancer intestinal cells (SIc). The aim was to obtain some of the missing data on particular in vitro activities of the test compound, to assist in identifying phytochemicals with scaffolds that possess a potent combination of bioactivities. They could subsequently be utilized in future chemotaxonomic investigation of antidiarrheal medicinal plants with their bioactive components considered for new chemotherapeutic agents against diarrheal infections and associated intestinal cancer diseases. Our results show that 8-hydroxyquinoline alkaloids and zinc pyrithione possess in vitro selective antibacterial properties against diarrheagenic bacteria comparable to ciprofloxacin and tetracycline, with additional in vitro antiproliferative activity against cancer intestinal cell lines. However, in contrary to antibiotics, these compounds generally possess increased cytotoxicity to normal intestinal cells.

## 2. Results

### 2.1. Antibacterial Activity

As far as the antibacterial activity of antibiotics against diarrheagenic strains is considered, ciprofloxacin and tetracycline exhibited strong growth-inhibitory effect ($\bar{x}$-MICs = 2 ± 4 and 4.8 ± 8 μg/mL, respectively), while chloramphenicol and ceftriaxone exhibited moderate growth-inhibitory activities ($\bar{x}$-MIC = 16.5 ± 34 and 61.8 ± 141 μg/mL, respectively). Regarding the significant degree of variation between MICs of these compounds, particular types of pathogenic strains were highly susceptible while some other species were distinctly more resistant. For example, all gram-negative diarrheagenic bacteria were highly susceptible to ciprofloxacin (MICs = 0.016–0.125 μg/mL) and ceftriaxone (MICs = 0.062–0.5 μg/mL). In contrast, their MICs produced against gram-positive pathogens were comparably higher; for the former ranging from 1 to 16 μg/mL and for the latter in the range of 4–512 μg/mL. At least half of the diarrheagenic bacteria were inhibited at the low MICs (1–4 μg/mL) by chloramphenicol and tetracycline, whereas the variations were particularly caused by the weak activities revealed against *Enterococcus faecalis* (MIC = 128 μg/mL) and *Clostridium perfringens* (32 μg/mL), respectively. At the low concentration (MICs = 0.5 μg/mL), tetracycline also inhibited *Clostridium difficile* and *Bacillus cereus.* Although metronidazole and vancomycin were generally inactive against diarrheagenic bacteria ($\bar{x}$-MIC = 651.4 ± 459 and 512.9 ± 404, respectively), they produced a strong inhibitory effect against both clostridial species tested (MICs = 0.5–8 μg/mL), whereas the former also exhibited strong activity against *Escherichia coli* (0.062 μg/mL). The synthetic analogs of phytochemicals, namely, zinc pyrithione, nitroxoline, and chloroxine, exhibited strong to moderate growth-inhibitory activity against all diarrheagenic bacteria ($\bar{x}$-MICs = 7.1 ± 4, 12 ± 10, and 24 ± 19 μg/mL, respectively). The activities of these compounds against particular pathogenic bacteria did not have significant differences, however, some of the strains were comparably more susceptible. Regarding that, *B. cereus*, *E. coli*, *Shigella flexneri*, and *Vibrio parahaemolyticus* were highly susceptible to zinc pyrithione (MICs = 1–4 μg/mL). The MIC of chloroxine against both *B. cereus* and *C. difficile* was 8 μg/mL. Nitroxoline exhibited strong growth-inhibitory activities (MICs = 2–4 μg/mL) against *B. cereus*, clostridial species, *E. coli*, and *S. flexneri*. Although 8-hydroxyquinoline did not produce significant antibacterial activity against the pathogens ($\bar{x}$-MIC = 224.4 ± 181), its growth-inhibitory activities were strong against *E. faecalis* (MIC = 4 μg/mL) and *Listeria monocytogenes* (MIC = 1 μg/mL).

Subsequently, the growth-inhibitory activities of different test compounds against probiotic strains were evaluated. Probiotic bacteria exhibited high susceptibility to chloramphenicol ($\bar{x}$*-*MIC = 6.2 ± 4 μg/mL) and medium susceptibility to tetracycline, nitroxoline, zinc pyrithione, ciprofloxacin, ceftriaxone, sanguinarine, and vancomycin ($\bar{x}$*-*MICs = 19.8 ± 19–47.8 ± 78 μg/mL). The MICs (2–4 μg/mL) of both chloramphenicol and vancomycin against bifidobacteria were similarly low, but the latter produced significantly lower activities against lactobacilli (MICs = 64–256 μg/mL) and *B. fragilis* (MIC = 32 μg/mL). With the exception of *Bifidobacterium breve* and *Bifidobacterium longum* ssp. *longum* (MICs = 8–32 μg/mL), the remaining bifidobacteria were also susceptible to ceftriaxone (MICs = 1–4 μg/mL). However, considering the MIC range (MICs = 0.5–32 μg/mL) shown against lactobacilli and the low activity against *B. fragilis* (MIC = 128 μg/mL), the variation of activities of this drug against probiotic bacteria is quite high. The exceptionally strong activities against *B. fragilis* were revealed by metronidazole and tetracycline (MICs = 0.5 μg/mL), whereas the MIC (4 μg/mL) of nitroxoline against this bacterium was the same as in the case of chloramphenicol. In general, berberine (MICs ≥ 32 μg/mL), ferron (MICs ≥ 64 μg/mL), and phenolic compounds (MICs ≥ 64 μg/mL) did not exhibit significant antibacterial activities against any of the 21 strains. The complete data on growth-inhibitory activities of test compounds against diarrheagenic and probiotic strains, including calculated mean values ($\bar{x}$*-*MIC) are presented in Table 1.

### 2.2. Cytotoxic Effect

Amongst the test compounds, only alkaloids and related structures exhibited strong cytotoxic activity, while other agents, especially antibiotics, exhibited moderate or no cytotoxic activity. Considering the antiproliferative effect of antibiotics on normal intestinal cells (FHs 74 Int), ceftriaxone, metronidazole, and vancomycin were not cytotoxic at all tested concentrations (IC_50_ and IC_80_ > 512 μg/mL), whereas tetracycline (IC_50_ = 14.7 ± 2.3 μg/mL; IC_80_ = 108.2 ± 5 μg/mL), chloramphenicol (IC_50_ = 30.7 ± 5.6 μg/mL; IC_80_ > 512 μg/mL), and ciprofloxacin (IC_50_ = 51.8 ± 27 μg/mL; IC_80_ = 129.5 ± 24 μg/mL) were moderately cytotoxic. In case of phytochemicals and their synthetic analogs, salicylic acid (IC_50_ = 73.2 ± 4.6 μg/mL; IC_80_ = 206.9 ± 69 μg/mL), ferron (IC_50_ = 22.6 ± 3.3 μg/mL; IC_80_ = 46 ± 2.2 μg/mL), and 8-hydroxyquinoline (IC_50_ = 10.7 ± 0.2 μg/mL; IC_80_ = 20.3 ± 2.4 μg/mL), revealed moderately cytotoxic effects against FHs 74 Int, whereas the other compounds were cytotoxic (IC_50_ values = 0.3 ± 0.1–1 ± 0.1 μg/mL; IC_80_ values = 0.5 ± 0.03–26.4 ± 0.8 μg/mL). Considering the antiproliferative effect on cancer intestinal cells, zinc pyrithione, 8-hydroxyquinoline, and sanguinarine were cytotoxic to HT29 (IC_50_ values = 0.6, 1.3, and 0.9 μg/mL, respectively) and Caco-2 (IC_50_ values = 0.7, 0.3 and 0.8 μg/mL, respectively) cells. Nitroxoline (IC_50_ = 1.1 μg/mL) and chloroxine (IC_50_ = 1.3 μg/mL) exhibited comparable cytotoxic activity against Caco-2 cells. Zinc pyrithione had the lowest $\bar{x}$*-*IC_50_ value (0.6 ± 0.05 μg/mL) against cancer cells, followed by 8-hydroxyquinoline (0.8 ± 0.5 μg/mL), sanguinarine (0.8 ± 0.05 μg/mL), nitroxoline (1.8 ± 0.8 μg/mL), and chloroxine (2.5 ± 1.2 μg/mL). Berberine ($\bar{x}$-IC_50_ = 12.2 ± 7 μg/mL), tannic acid ($\bar{x}$-IC_50_ = 31.7 ± 4 μg/mL), and ferron ($\bar{x}$*-*IC_50_ = 70.8 ± 16 μg/mL), produced moderate cytotoxic activity, while salicylic acid and bismuth subsalicylate ($\bar{x}$*-*IC_50_ ≥ 253.6 ± 208 μg/mL) did not exhibit significant cytotoxic activity against cancer cells. At relatively high concentrations, some antibiotics exhibited antiproliferative activity against cancer cells, namely: ciprofloxacin, tetracycline, and chloramphenicol ($\bar{x}$*-*IC_50_ = 100.1 ± 30–355.2 ± 84 μg/mL). The complete data on the antiproliferative activities of test compounds against normal and cancer intestinal cells, including calculated mean values for the latter ($\bar{x}$*-*IC_50_ and $\bar{x}$*-*IC_80_), are presented in Table 1.

### 2.3. Selective Toxicity

**Figure 1:**
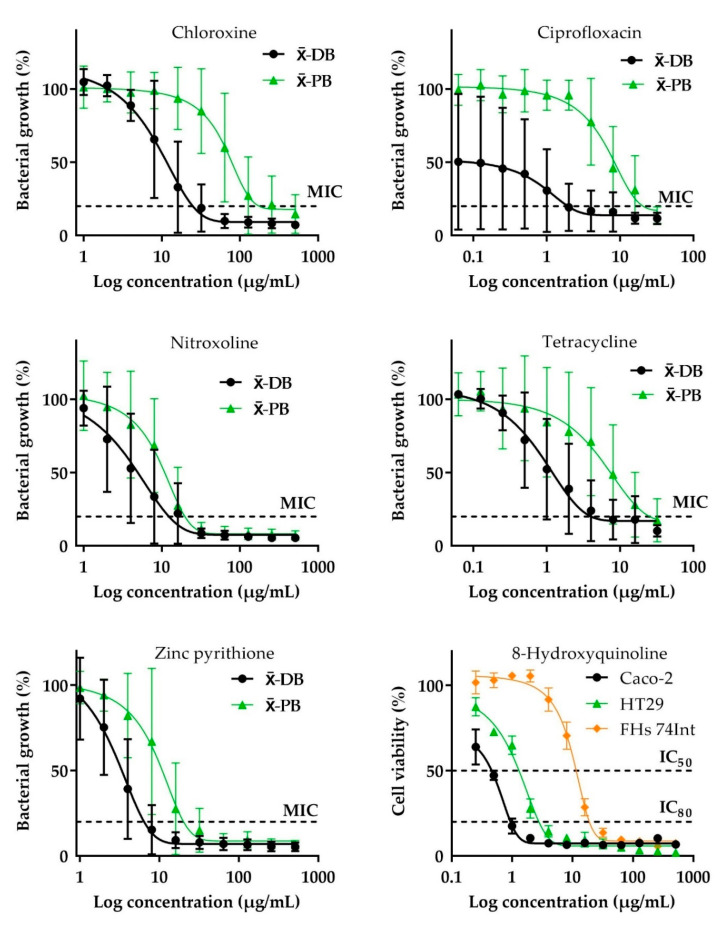
Selective concentration-dependent effect of chloroxine, ciprofloxacin, nitroxoline, tetracycline, and zinc pyrithione on the growth of diarrheagenic and probiotic bacteria and of 8-hydroxyquinoline on intestinal normal and cancer cells proliferation in vitro. MIC: minimum inhibitory concentration; IC_50_: half maximal inhibitory concentration; IC_80_: 80% inhibitory concentration of proliferation. $\bar{x}$ -DB: mean MIC for 12 diarrheagenic bacteria, $\bar{x}$ -PB: mean MIC for 9 probiotic bacteria; Caco-2 and HT29: intestinal cancer cells; Fhs74 Int: intestinal normal cells.

The selective antibacterial activities against the pathogens with relatively lower activity against probiotic strains (SIb values range from 0.2–1.1) was revealed by most of the agents exhibiting strong to moderate inhibitory effects on diarrheagenic bacteria, namely: nitroxoline, zinc pyrithione, tetracycline, chloroxine, and ciprofloxacin. In contrast, chloramphenicol and ceftriaxone were more toxic to probiotic strains (SIbs = −0.4 for both). Although the antibacterial activity of berberine, ferron, phenolic compounds, and sanguinarine was in cases of both diarrheagenic and probiotic strains generally insignificant, the results show that these agents were rather toxic to the latter (SIb values range from −1 to −0.03). Due to the minor cytotoxicity revealed against FHs 74 Int, none of the antibiotics exhibited an increased toxicity to normal intestinal cells at the inhibitory concentrations active against diarrheagenic bacteria (SIa values = 0.2–1.8), especially ciprofloxacin and chloramphenicol. In contrast, all of the phytochemicals and their synthetic analogs revealed cytotoxicity to normal intestinal cells at the concentrations they were generally inactive against diarrheagenic bacteria (SIa values range from −1.9 to −0.7). Only 8-hydroxyquinoline (SIc = 1.1) and sanguinarine (SIc = 0.1) exhibited selective antiproliferative activity against cancer cells with the decreased cytotoxic effect on normal intestinal cells. Except these two, other tested compounds were more toxic to normal than to cancer intestinal cells (SIcs = from −1.5 to −0.3), or in the case of ceftriaxone, metronidazole, and vancomycin, they did not show any selectivity (SIcs = 0), as they did not inhibit cell lines at any concentration tested. The data on selective toxicities, including all calculated SI values are presented in Table 1. The curves of in vitro selective concentration-dependent effect of ciprofloxacin, chloroxine, nitroxoline, tetracycline, and zinc pyrithione on the growth of diarrheagenic and probiotic bacteria and of 8-hydroxyquinoline on intestinal normal and cancer cells proliferation are shown in Figure 1.

### 2.4. Principal Component Analysis (PCA)

**Figure 2:**
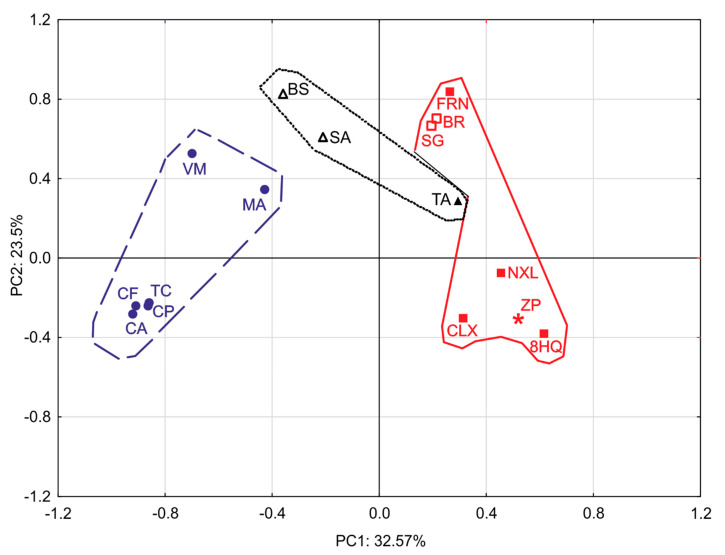
Principal component analysis of antibacterial and cytotoxic activities of phytochemicals, their synthetic analogues, and antibiotics against intestinal bacteria and cells in vitro. BR: berberine, SG: sanguinarine, 8HQ: 8-hydroxyquinoline, CLX: chloroxine, NXL: nitroxoline, FRN: ferron, ZP: zinc pyrithione, SA: salicylic acid, BS: bismuth subsalicylate, TA: tannic acid, CF: ceftriaxone, CP: ciprofloxacin, MA: metronidazole, VM: vancomycin, CA: chloramphenicol, TC: tetracycline. Antibiotics [**-------**, **●**]; Phenolic compounds [**^…….^**, poly- (▲), simple (**∆**)]; Alkaloids and related structures [**^_____^**, benzylisoquinolines (**□**), 8-hydroxyquinolines (**■**), metal-pyridine derivative complex (*).

The correlation between biological activities and chemical structures of the tested compounds and their groups (antibiotics, phenolic compounds, alkaloids, and related structures) was analyzed using PCA (Figure 2). Although the compounds were distributed equally in all quadrants, detailed analysis revealed specific patterns. The closest correlation was observed in the lower-left quadrant between four antibiotics (ceftriaxone, ciprofloxacin, chloramphenicol, and tetracycline), which indicated that these antibiotics exhibited strong growth-inhibitory activity against diarrheagenic and probiotic strains but did not exhibit distinct cytotoxic activity against normal and cancer intestinal cells. The second highest correlation observed in the lower right quadrant indicated that zinc pyrithione and 8-hydroxyquinolines (excluding ferron) exhibited selective antibacterial activity against diarrheagenic strains along with strong to moderate cytotoxic activity against both types of tested cell lines. The correlation observed in the upper right quadrant indicated that tannic acid, benzylisoquinoline alkaloids, and ferron exhibited moderate to no antipathogenic effect (with negative SIbs) and overall moderate to strong cytotoxic activity. The upper left quadrant contains the remaining antibiotics (metronidazole and vancomycin) and both simple phenols with minimal correlation. These agents exhibited moderate to no growth-inhibiting activity against diarrheagenic strains and practically no cytotoxic activity. Whereas all alkaloids are distributed in lower and upper right quadrants indicating their capability to reveal any type of the tested bioactivities, phenols are spread in the right and left upper quadrants which shows their lack of significant antibacterial activity but a certain degree of cytotoxicity. In contrast, antibiotics are concentrated in the lower-left quadrant slightly overlapping the upper left one, therefore they usually display a significant antibacterial effect which is rarely accompanied by cytotoxicity.

## 3. Discussion

The in vitro growth-inhibitory properties of the tested antibiotics have previously been reported for a number of diarrheagenic and probiotic bacteria. However, the bacterial strains tested as well as the methods with criteria used for antimicrobial activity assessment vary frequently among the previous studies. Moreover, the studies reporting the in vitro susceptibilities of probiotic bacteria deal more with clinical isolates [23], and less with standard strains [24]. The present study, therefore, provides the data on in vitro selective antibacterial activities of these antibiotics that can be fairly compared with the same data obtained for phytochemicals and their synthetic analogs. We suggest that the reason behind the increased resistance of probiotic strains differ for particular compounds that showed a selective antipathogenic effect. The growth-inhibitory activities of fluoroquinolones against Gram-positive bacteria are reported to be lower than those against Gram-negative bacteria [15]. Consistent with this finding, bifidobacteria and lactobacilli (Gram-positive) were generally less susceptible to ciprofloxacin than Gram-negative diarrheagenic bacteria that predominate over Gram-positive pathogens in this study. The decreased susceptibility of bifidobacteria to tetracycline might be caused by the presence of specific antibiotic resistance genes [25]. Similar to other third-generation cephalosporins [26], the growth-inhibitory activity of ceftriaxone against Gram-negative bacteria was higher than that against Gram-positive bacteria. However, as a result of significant resistance of the tested Gram-positive pathogens and susceptibility of bifidobacteria, ceftriaxone showed increased toxicity to probiotic strains. The growth-inhibitory activities of some alkaloids and related structures were comparable with those of antibiotics. The antibacterial activity of 8-hydroxyquinoline alkaloids is mediated through the chelation of metals that function as co-factors in various enzymes, which results in the inhibition of RNA synthesis. We suggest that probiotic strains (mainly bifidobacteria) are more resistant to 8-hydroxyquinolines as they are able to sequester iron from the environment [10]. The selective antibacterial activity of 8-hydroxyquinoline against diarrheagenic pathogens seems to be enhanced with chlorine halogenation or by the presence of a nitro group and decreased with iodine halogenation and the presence of a sulfo group, as respectively observed for chloroxine, nitroxoline, and ferron in our study. The in vitro selective anticlostridial effect of 8-hydroxyquinoline with increased resistance of bifidobacteria was previously described in studies of Novakova et al. [10,27,28], Skrivanova et al. [29], and Kim et al. [30]. However, data on its in vitro growth-inhibitory effects against a broader selection of diarrheagenic bacteria are limited. The present study also provides new data on in vitro antibacterial activities of chloroxine against diarrheagenic bacteria in addition to those previously published [31,32]. It has been reported that Endiaron, a chloroxine-containing antimicrobial product used for infectious diarrhea, exhibits antimicrobial activity against the pathogens and does not affect the host indigenous microbiota [11], which is in agreement with the increased resistance of probiotic bacteria described in the present study. Interestingly, the antipathogenic activity of nitroxoline, used to treat urinary tract infections, was higher than that of chloroxine. However, the antibacterial selectivity of nitroxoline against diarrheagenic strains was lower. Out of the intestinal bacteria tested herein, there are only data on in vitro inhibitory effects of nitroxoline against *E. coli* and *E. faecalis* that have been reported before [33]. In spite of zinc pyrithione being only used topically for dermatological infections [7], in relation to the plant compounds and their synthetic analogs in this study, it exhibited the highest growth-inhibitory activity against diarrheagenic bacteria with lowered toxicity to probiotic bacteria. According to our best knowledge, this is the first report on in vitro selective antibacterial activities of zinc pyrithione on intestinal diarrheagenic and probiotic bacteria. Although there is limited knowledge on the mechanism underlying the antibacterial activity of zinc pyrithione, the mechanism may be similar to that of 8-hydroxyquinolines [34]. The weak antimicrobial activities of phenols against diarrheagenic strains are consistent with those reported in previous studies. The effectiveness of phenols in infectious diarrhea may be based on other mechanisms, such as astringent, mucosa-protective, and anti-inflammatory properties, or inhibition of pathogenic enterotoxins [7]. Although clinical studies on extensively used phytochemical berberine have reported comparably higher efficiency than certain antibiotics (e.g., chloramphenicol) [35], our results did not show its significant in vitro antibacterial activity. A possible reason for this discordance is that berberine rather neutralizes diarrheagenic action of bacteria by inhibiting their enterotoxins, as described by Sack and Froelich [36]. The MICs of both benzylisoquinoline alkaloids against some diarrheagenic bacteria reported in this study were higher than those reported in previous studies. This may be because the inoculum density used in this study was higher than that used in previous studies [7,37].

The mechanism underlying the antiproliferative activity of some antibiotics, such as ciprofloxacin, tetracycline, and chloramphenicol, may be similar to that underlying antimicrobial activity [14,38,39]. Previous studies have evaluated the antiproliferative activity of chemicals derived from ciprofloxacin and tetracycline against cancer cells and suggested their applications in cancer therapy [14,40]. Consistent with the results of this study, previous studies have revealed that other antibiotics are not cytotoxic to eukaryotic cells [41,42,43]. The antitumor activities of some plant compounds and their synthetic analogs have been investigated previously. In the case of 8-hydroxyquinoline and its derivatives, the interaction with metal ions, namely copper and iron, and their transportation into cells has been reported as crucial for its antiproliferative activity [44]. Freitas et al. [45] reported that 8-hydroxyquinoline derivatives with potent anticancer potential often contain halogen substituents. However, in the present study, nitroxoline exhibited stronger antiproliferative activity against cancer cells than chloroxine and ferron. Previous studies have reported that the underlying mechanism of antitumor activity of zinc pyrithione and sanguinarine involves the inhibition of proteasomal deubiquitinases and microtubule depolymerization, respectively [19,22]. All of the above-mentioned alkaloids and related structures revealed increased toxicity to normal intestinal cells in comparison with their antipathogenic effect, which limits their applications in treating bacterial diarrhea. Although there are no studies reporting oral toxicity or toxicity to the digestive system from berberine, chloroxine, and nitroxoline, we suggest that their safety profile should be further examined and the potential protective role of indigenous gut microbiota against these cytotoxic chemicals should be more deeply studied. Zinc pyrithione, 8-hydroxyquinoline, and sanguinarine are not part of any product intended for internal use. Hence, their dose-dependent toxicological profile and oral safety must be carefully elucidated before any consideration for their application for treating infectious diarrhea associated with intestinal cancer [46].

## 4. Materials and Methods

### 4.1. Chemicals

**Figure 3:**
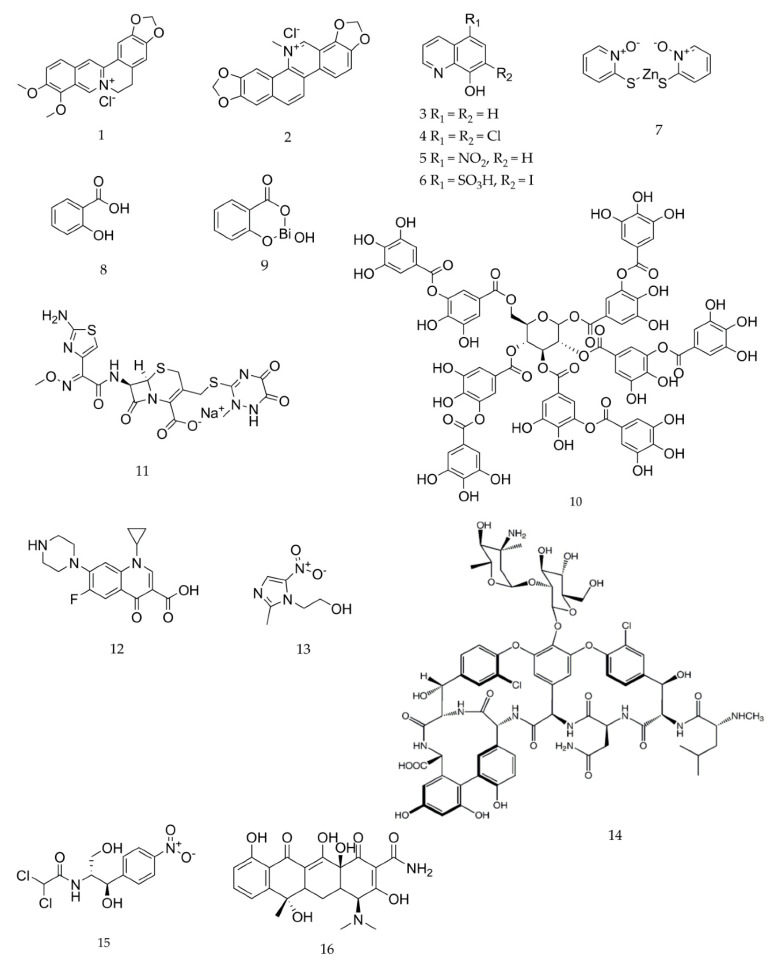
The chemical structures of the tested phytochemicals, their synthetic analogs, and antidiarrheal antibiotics. **1**. berberine chloride, **2**. sanguinarine chloride, **3**. 8-hydroxyquinoline, **4**. chloroxine, **5**. nitroxoline, **6**. ferron, **7**. zinc pyrithione, **8**. salicylic acid, **9**. bismuth subsalicylate, **10**. tannic acid, **11**. ceftriaxone sodium, 12. ciprofloxacin, **13**. metronidazole, **14**. vancomycin hydrochloride, **15.** chloramphenicol, **16**. tetracycline.

Phytochemicals (berberine chloride, 8-hydroxyquinoline, salicylic acid, tannic acid, and sanguinarine chloride) and their synthetic analogs [chloroxine (5,7-dichloroquinolin-8-ol), nitroxoline (5-nitroquinolin-8-ol), ferron (7-iodo-8-hydroxyquinoline-5-sulfonic acid), bismuth subsalicylate, and zinc pyrithione], as well as antibiotics (ceftriaxone sodium, ciprofloxacin, chloramphenicol, metronidazole, tetracycline, and vancomycin hydrochloride), used in this study were purchased from Sigma-Aldrich (Prague, Czech Republic). Dimethyl sulfoxide (DMSO) (Sigma-Aldrich, Prague, Czech Republic) was used to prepare the stock solutions of all test compounds, except those of metronidazole, salicylic acid, vancomycin, and zinc pyrithione, which were prepared using distilled water. Stock solutions of chloramphenicol, tannic acid, and tetracycline were prepared using 96% ethanol (Sigma-Aldrich, Prague, Czech Republic). The chemical structures of individual compounds tested are shown in Figure 3.

### 4.2. Bacterial Strains and Growth Media

The intestinal bacterial type strains were obtained from the American Type Culture Collection (ATCC, Rockville, MD, USA), Czech Collection of Microorganisms (CCM, Brno, Czech Republic), German Collection of Microorganisms and Cell Cultures (DSMZ, Braunschweig, Germany), and National Collection of Type Cultures (NCTC, London, UK). In accordance with the diversity of diarrheagenic Gram-positive and Gram-negative bacteria responsible for globally distributed foodborne, waterborne, and nosocomial infections [3,47], the following 12 strains were used in this study: *B. cereus* (ATCC 14579), *C. difficile* (DSMZ 12056), *C. perfringens* (DSMZ 11778), *E. faecalis* (ATCC 29212), *E. coli* (ATCC 25922), *E. coli* 0175:H7 (NCTC 12900), *L. monocytogenes* (ATCC 7644), *S. flexneri* (ATCC 12022), *Salmonella enterica* ssp. *enterica* serovar Enteritidis (ATCC 13076), *S. enterica* ssp. *enterica* serovar Typhimurium (ATCC 14028), *V. parahaemolyticus* (ATCC 17802), and *Yersinia enterocolitica* (ATCC 9610). The following nine bacterial strains, which belong to three predominant bacterial phyla in the human gut and exhibit probiotic functions [8,9], were used in this study: *Bacteroides fragilis* (ATCC 25285), *Bifidobacterium adolescentis* (DSMZ 20087), *Bifidobacterium animalis* spp. *lactis* (DSMZ 10140), *Bifidobacterium bifidum* (ATCC 29521), *B. breve* (ATCC 15700), *B. longum* ssp. *longum* (DSMZ 20219), *Lactobacillus casei* (DSMZ 20011), *Lactobacillus reuteri* (CCM 3625), and *Lactobacillus rhamnosus* (CCM 7091). As the maintenance and growth medium, Mueller–Hinton broth (Oxoid, Basingstoke, UK) was used for bacteria that grow aerobically (*E. faecalis* supp. 1% glucose, *V. parahaemolyticus* supp. 3% NaCl). The anaerobic bacteria (clostridia and bifidobacteria), including facultative species (lactobacilli), were cultured in Wilkins–Chalgren broth (Oxoid, Basingstoke, UK) supplemented with 5 g/L soya peptone and 0.5 g/L cysteine.

### 4.3. Cell Cultures

One representative normal intestinal cell line (FHs 74 Int (ATCC CCL 241)) and two cancer intestinal cell lines (Caco-2 (ATCC HTB 37) and HT29 (ATCC HTB 38)) were purchased from ATCC (Rockville, MD, USA). Normal cells were cultured in Hybri-Care medium supplemented with 10% fetal bovine serum, 1% sodium bicarbonate, 1% non-essential amino acids, 30 ng/mL of epidermal growth factor, and 1% penicillin-streptomycin solution (10,000 units/mL and 100 mg/mL, respectively). The cancer cells were cultured in Dulbecco’s modified Eagle’s medium (DMEM) supplemented with 1% sodium pyruvate, 10% fetal bovine serum, 1% sodium bicarbonate, 1% non-essential amino acids, and 1% penicillin-streptomycin solution (10,000 units/mL and 100 mg/mL, respectively) (all purchased from Sigma-Aldrich, Prague, Czech Republic). The cultures were incubated at 37 °C and 5% CO_2_. The culture medium was replaced every 2–3 days and cells were passaged every 7 days.

### 4.4. Antibacterial Assay

The growth-inhibitory activities of the test compounds against aerobic and anaerobic bacterial strains were evaluated by the broth microdilution method using 96-well microtiter plates, following the protocols of CLSI guidelines [48] and Hecht et al. [49], respectively. Prior to testing, the strains were sub-cultured in the appropriate media at 37 °C for 24 h. Obligate anaerobes and lactobacilli were cultured for 48 h using Whitley A35 Anaerobic Workstation (Don Whitley Scientific, Bingley, UK). The anaerobic conditions were created by the supply of a standard anaerobic gas mixture of 10% H_2_, 10% CO_2_, and 80% N_2_ (Linde Gas, Prague, Czech Republic). Test agents were diluted 2-fold in appropriate growth media using the Freedom EVO 100 automated pipetting platform (Tecan, Männedorf, Switzerland) or multichannel pipette (Eppendorf, Hamburg, Germany) (initial concentration of 512 μg/mL). After the bacterial cultures reached an inoculum density of 1.5 × 10^8^ CFU/mL by 0.5 McFarland standard using Densi-La-Meter II (Lachema, Brno, Czech Republic), the 96-well plates were inoculated (5 μL/well). The plates containing the volatile compound, 8-hydroxyquinoline, were covered using EVA capmats (Micronic, Lelystad, Netherlands) after inoculation to prevent evaporation [50]. Bacterial cultures in microplates were incubated by employing the same protocols as used for their cultivation prior to the test. The optical density of the cultures was measured at 405 nm (OD_450 nm_) using a Cytation 3 Imaging Reader (BioTek, Winooski, VT, USA) before and after the growth. The lowest concentration (μg/mL) of test compounds at which the bacterial growth was inhibited by ≥80% was defined as MIC. All tests were performed as three independent experiments each carried out in triplicate. The data are presented as median/mode. As a result of experiments performed without dissolved test compounds, DMSO and 96% ethanol (both from Sigma-Aldrich, Prague, Czech Republic) did not inhibit bacterial growth of any strain at the tested concentrations (≤1%).

### 4.5. Cytotoxicity Assay

The antiproliferative activities of test compounds against normal and cancer intestinal lines were evaluated using the modified thiazolyl blue tetrazolium bromide (MTT) cytotoxicity assay developed by Mosmann et al. [51]. The cancer (2.5 × 10^3^) and normal intestinal (2.5 × 10^5^) cells were seeded in a 96-well microtiter plate for 24 h. Cells were incubated with two-fold serially diluted test compounds (0.25–512 μg/mL) for 72 h. Plates containing 8-hydroxyquinoline were covered using EVA capmats. Next, the cells were incubated with MTT reagent (1 mg/mL) (Sigma-Aldrich, Prague, Czech Republic) in DMEM or Hybri-Care medium for an additional 2 h at 37 °C and 5% CO_2_. The medium with MTT was removed and the intracellular formazan product was dissolved in 100 μL of DMSO. The absorbance was measured at 555 nm using a Tecan Infinite M200 spectrometer (Tecan, Männedorf, Switzerland), and the percentage of viability was calculated when compared to an untreated control. Antiproliferative activity of the test compounds was represented as IC_50_ (μg/mL). Three independent experiments (two replicates each) were performed for every test. Data are presented as mean ± standard deviation. DMSO was used as a positive control at the highest concentration with and without EVA capmats. The solvents did not affect the viability of normal and cancer intestinal cell lines at the tested concentration (≤1%).

### 4.6. Calculations and Statistics

For comparison of microbiological and toxicological data, IC_80_ was calculated as equivalent to the MIC endpoint, defined as 80% bacterial growth inhibition [52]. Subsequently, $\bar{x}$*-*MIC, $\bar{x}$*-*IC_50_, and $\bar{x}$*-*IC_80_ values (±standard deviations) were calculated to quantify the inhibitory activity of test compounds against diarrheagenic/probiotic bacteria and cancer cells, respectively. After that, SIa (normal intestinal cells/ diarrheagenic strains), SIb (probiotic/diarrheagenic strains), and SIc (normal/cancer intestinal cells) was calculated using the formulas below.

SIa = log (X_1_/Y_1_),

SIb = log (X_2_/Y_1_)

SIc = log (X_3_/Y_2_)

where: X_1_ = IC_80_ against FHs 74 Int; X_2_ = $\bar{x}$*-*MIC against probiotic strains; X_3_ = IC_50_ against FHs 74 Int; Y_1_ = $\bar{x}$*-*MIC against diarrheagenic strains; Y_2_ = $\bar{x}$*-*IC_50_ against cancer intestinal cells. The SI values > 0 and <0 indicate selective toxicity against diarrheagenic strains/cancer cell lines and probiotic strains/normal cell lines, respectively.

The correlation between the combination of activities revealed by test compounds and their chemical classes was analyzed using PCA with Statistica 13 software [53]. All data for particular activities were grouped into four types of targets (cancer cells, diarrheagenic strains, normal cells, and probiotic strains) using MIC and IC_80_ values. There was no adjustment of PCA parameters for this analysis. For the calculation of each $\bar{x}$*-*MIC, $\bar{x}$*-*IC_50_, $\bar{x}$*-*IC_80_ and for PCA, values greater than the maximum tested concentration (512 μg/mL) were replaced with 1024 μg/mL.

## 5. Conclusions

In summary, ciprofloxacin, 8-hydroxyquinoline alkaloids (chloroxine and nitroxoline), tetracycline, and zinc pyrithione exhibited a significant selective growth-inhibitory activity against diarrheagenic bacteria with lowered toxicity to probiotic bacteria in vitro. 8-Hydroxyquinoline, chloroxine, nitroxoline, sanguinarine, and zinc pyrithione also exhibited a strong cytotoxic effect, whereas the antiproliferative action of 8-hydroxyquinoline and sanguinarine were selective to cancer intestinal cells. These findings indicate that 8-hydroxyquinoline alkaloids and metal-pyridine derivative complexes are chemical structures with promising bioactive properties in terms of in vitro selective antibacterial and anticancer activities which could be utilized in future chemotaxonomic investigation of antidiarrheal medicinal plants and their bioactive components. These could be further investigated as possible new chemotherapeutic agents against diarrheal infections and associated intestinal cancer diseases. However, in vivo studies on the toxicity of these compounds with more complex animal models will be needed before their consideration to be used for this purpose.

## Figures and Tables

**Table 1:** In vitro selective inhibitory activities of phytochemicals, their synthetic analogs, and antibiotics against intestinal bacteria and cells.

Cultures Tested			Alkaloids							Phenolic Compounds			Antibiotics					
			BR	SG	8HQ	CLX	NXL	FRN	ZP	SA	BS	TA	CF	CP	MA	VM	CA	TC
Bacterial strain/MIC (μg/mL)		BC	- ^a^	128	512	8	4	512	4	-	512	512	128	1	128	256	8	0.5
		CD	-	64	128	8	2	64	8	256	128	-	64	16	0.5	2	4	0.5
		CP	256	128	128	16	4	64	8	-	512	512	4	1	8	1	4	32
		EF	-	32	4	16	32	-	8	-	-	-	512	2	-	-	128	4
		EC	-	256	128	16	4	-	4	-	-	-	0.062	0.062	0.062	-	4	1
		ECS	-	128	256	64	32	-	8	-	-	512	0.5	0.016	-	512	8	4
		LM	-	16	1	32	16	512	8	-	256	-	32	4	512	8	8	2
		SF	-	64	128	16	2	-	1	-	-	-	0.5	0.016	-	-	4	2
		SE	-	256	256	64	16	-	8	-	-	-	0.25	0.031	-	512	4	4
		ST	512	512	512	16	16	-	8	-	512	-	0.25	0.031	-	512	8	4
		VP	512	32	128	16	8	256	4	-	-	128	0.125	0.062	-	256	2	1
		YE	-	256	512	16	8	-	16	-	-	512	0.25	0.125	-	-	16	2
		$\bar{x}$*-*DB ± SD	874.7 ± 256	156 ± 137	224.4 ± 181	24 ± 19	12 ± 10	714.7 ± 388	7.1 ± 4	960 ± 212	757.3 ± 332	778.7 ± 307	61.8 ± 141	2 ± 4	651.4 ± 459	512.9 ± 404	16.5 ± 34	4.8 ± 8
		BF	-	32	32	32	4	128	8	128	32	-	128	8	0.5	32	4	0.5
		BA	128	16	512	128	16	256	16	-	32	-	1	8	64	2	4	64
		BLC	32	32	-	512	32	512	16	128	64	-	2	32	32	2	4	32
		BBF	64	32	512	-	16	512	8	-	64	-	4	16	-	4	4	16
		BB	64	32	-	64	32	512	16	-	64	256	32	64	32	4	4	16
		BL	32	64	512	64	16	256	16	512	64	128	8	16	8	2	4	2
		LC	64	32	-	128	32	256	16	512	128	512	32	32	-	256	16	8
		LR	-	32	-	128	16	512	64	512	64	512	0.5	32	256	64	8	32
		LRM	64	64	-	128	16	512	32	512	128	-	32	4	-	64	8	8
		$\bar{x}$*-*PB ± SD	277.3 ± 400	37.3 ± 15	743.1 ± 343	245.3 ± 306	20 ± 9	384 ± 148	21.3 ± 16	597.3 ± 336	71.1 ± 33	725.3 ± 352	26.6 ± 38	23.6 ± 18	384.9 ± 458	47.8 ± 78	6.2 ± 4	19.8 ± 19
Cell line (μg/mL)	IC_50_ ± SD	HT29	5 ± 1	0.9 ± 0.2	1.3 ± 0.3	3.7 ± 0.3	2.6 ± 0.3	86.3 ± 12	0.6 ± 0.01	-	461.9 ± 17	35.9 ± 4.9	-	130.3 ± 13	-	-	271.1 ± 1	392.9± 20
		Caco-2	19.4 ± 2.9	0.8 ± 0.1	0.3 ± 0.1	1.3 ± 0.04	1.1 ± 0.03	55.2 ± 4.8	0.7 ± 0.2	-	45.3 ± 5	27.6 ± 1.5	-	69.9 ± 4.9	-	-	439.3 ± 4	70.4 ± 15
		$\bar{x}$*-*CC ± SD	12.2 ± 7	0.8 ± 0.05	0.8 ± 0.5	2.5 ± 1.2	1.8 ± 0.8	70.8 ± 16	0.6 ± 0.05	-	253.6 ± 208	31.7 ± 4	-	100.1 ± 30	-	-	355.2 ± 84	231.6± 161
		FHs 74 Int	1 ± 0.1	1 ± 0.1	10.7 ± 0.2	0.5 ± 0.02	0.4 ± 0.05	22.6 ± 3.3	0.3 ± 0.1	73.2 ± 4.6	8.7 ± 1.3	5.9 ± 1.2	-	51.8 ± 27	-	-	30.7 ± 5.6	14.7 ± 2.3
	IC_80_ ± SD	HT29	42.1 ± 7.3	1.8 ± 0.4	4.8 ± 2.8	5 ± 0.7	3.7 ± 0.4	149.4 ± 6	0.7 ± 0.01	-	-	43 ± 2.2	-	-	-	-	-	-
		Caco-2	78.9 ± 0.3	1.5 ± 0.1	0.9 ± 0.1	5.9 ± 0.7	5 ± 1.5	139.9 ± 26	0.7 ± 0.01	-	454.9 ± 43	-	-	-	-	-	-	-
		$\bar{x}$*-CC* ± SD	*60.5 ± 18.4*	*1.6 ± 0.2*	*2.9 ± 2*	*5.4 ± 0.5*	*4.4 ± 0.7*	*144.7 ± 4.8*	*0.7 ± 0*	*-*	*740 ± 285*	*534 ± 491*	*-*	*-*	*-*	*-*	*-*	*-*
		FHs 74 Int	26.4 ± 0.8	1.9 ± 0.2	20.3 ± 2.4	2 ± 0.1	0.8 ± 0.05	46 ± 2.2	0.5 ± 0.03	206.9 ± 69	31.4 ± 6.6	10.2 ± 2.4	-	129.5 ± 24	-	-	-	108.2 ± 5
SI		(a)	−1.5	−1.9	−1	−1.1	−1.2	−1.2	−1.1	−0.7	−1.4	−1.9	1.2	1.8	0.2	0.3	1.8	1.4
		(b)	−0.5	−0.6	0.5	1	0.2	−0.3	0.5	−0.2	−1	−0.03	−0.4	1.1	−0.2	−1	−0.4	0.6
		(c)	−1.1	0.1	1.1	−0.7	−0.6	−0.5	−0.4	−1.1	−1.5	−0.7	0	−0.3	0	0	−1.1	−1.2

MIC: minimum inhibitory concentration; IC_50_: half maximal inhibitory concentration; IC_80_: 80% inhibitory concentration of proliferation; SD. standard deviation. ^a^ Not active (MIC/IC_50/80_ > 512 μg/mL, the value 1024 μg/mL was used for average calculation). BR: berberine, SG: sanguinarine, 8HQ: 8-hydroxyquinoline, CLX: chloroxine, NXL: nitroxoline, FRN: ferron, ZP: zinc pyrithione, SA: salicylic acid, BS: bismuth subsalicylate, TA: tannic acid, CF: ceftriaxone, CP: ciprofloxacin, MA: metronidazole, VM: vancomycin, CA: chloramphenicol, TC: tetracycline. BC: *Bacillus cereus*, CD: *Clostridium difficile*, CP: *Clostridium perfringens*, EF: *Enterococcus faecalis*, EC: *Escherichia coli*, ECS: *E. coli* 0175:H7, LM: *Listeria monocytogenes*, SF: *Shigella flexneri*, SE: *Salmonella* Enteritidis, ST: *Salmonella* Typhimurium, VP: *Vibrio parahaemolyticus*, YE: *Yersinia enterocolitica*, BF: *Bacteroides fragilis*, BA: *Bifidobacterium adolescenti*s, BLC: *Bifidobacterium animalis* spp. *lactis*, BBF: *Bifidobacterium bifidum*, BB: *Bifidobacterium breve*, BL: *Bifidobacterium longum* ssp. *longum*, LC: *Lactobacillus casei*, LR: *Lactobacillus reuteri*, LRM: *Lactobacillus rhamnosus*. $\bar{x}$ -DB: mean MIC for diarrheagenic bacteria, $\bar{x}$ -*PB*: mean MIC for probiotic bacteria, $\bar{x}$ *-CC*: mean IC_50/80_ for intestinal cancer cells, FHs 74 Int (intestinal normal cells), SD: standard deviation. SI (Selective Index): (**a**) normal cells/diarrheagenic bacteria, (**b**) probiotic bacteria/diarrheagenic bacteria, (**c**) normal cells/cancer cells.
